# Composition and Structure Progress of the Catalytic Interface Layer for Bipolar Membrane

**DOI:** 10.3390/nano12162874

**Published:** 2022-08-21

**Authors:** Di Zhao, Jinyun Xu, Yu Sun, Minjing Li, Guoqiang Zhong, Xudong Hu, Jiefang Sun, Xiaoyun Li, Han Su, Ming Li, Ziqi Zhang, Yu Zhang, Liping Zhao, Chunming Zheng, Xiaohong Sun

**Affiliations:** 1 School of Chemical Engineering, Tianjin Key Laboratory of Green Chemical Technology and Process Engineering, State Key Laboratory of Separation Membrane and Membrane Processes, Tiangong University, Tianjin 300387, China; 2 School of Materials Science and Engineering, Key Laboratory of Advanced Ceramics and Machining Technology, Ministry of Education, Tianjin University, Tianjin 300072, China; 3 Beijing Key Laboratory of Diagnostic and Traceability Technologies for Food Poisoning, Beijing Center for Disease Prevention and Control, Beijing 100013, China; 4 Advanced Materials Research Laboratory, CNOOC Tianjin Chemical Research and Design Institute, Tianjin 300131, China

**Keywords:** bipolar membrane, water dissociation, interface layer, catalyst, organic material, inorganic materials

## Abstract

Bipolar membranes, a new type of composite ion exchange membrane, contain an anion exchange layer, a cation exchange layer and an interface layer. The interface layer or junction is the connection between the anion and cation exchange layers. Water is dissociated into protons and hydroxide ions at the junction, which provides solutions to many challenges in the chemical, environmental and energy fields. By combining bipolar membranes with electrodialysis technology, acids and bases could be produced with low cost and high efficiency. The interface layer or junction of bipolar membranes (BPMs) is the connection between the anion and cation exchange layers, which the membrane and interface layer modification are vital for improving the performance of BPMs. This paper reviews the effect of modification of a bipolar membrane interface layer on water dissociation efficiency and voltage across the membrane, which divides into three aspects: organic materials, inorganic materials and newly designed materials with multiple components. The structure of the interface layer is also introduced on the performance of bipolar membranes. In addition, the remainder of this review discusses the challenges and opportunities for the development of more efficient, sustainable and practical bipolar membranes.

## 1. Overview of Bipolar Membranes

Bipolar membranes (BPMs) are one kind of special ion exchange membranes, which contain an anion exchange layer (AEL), a cation exchange layer (CEL) and an intermediate layer (IL). The intermediate layer is the junction of the anion and cation exchange layers. When the applied reverse bias potential causes the potential of the bipolar membranes reach or exceed 0.83 V, a large electric field is generated in the interface layer [1] to split water molecules into H^+^ and OH^−^. The generated H^+^ and OH^−^ pass through the cation-exchange membrane and anion-exchange membrane of the bipolar membrane, together with cation and anion exchange membranes in an electrodialytic cell arrangement, forming acids and bases in the adjacent two compartments. The hydrolysis rate of the interface layer applied with a DC electric field is much higher than that of the general case [2,3]. During the electrolysis process, the water consumed by the dissociation of the water in the interface layer of bipolar membranes can be replenished in time by the water in the electrolyte on both sides of the membranes. Unlike the reactions on the electrodes during electrolysis, this process does not generate any gas [4,5] and can obviously reduce energy consumption.

This unique advantage of bipolar membranes has led to an increasing interest in it. The improvement of the bipolar membrane interfacial layer material accelerates the rate of water dissociation in the interface layer, decreases the hydrolysis voltage and reduces energy consumption. The effect of the performance of synthetic bipolar membranes can be explained by applying them in electrolysis experiments on brine in electrodialysis. In recent years, researchers have devoted much effort in regard to the interface layer materials for this purpose. Eswaraswamy et al. introduced a nanocomposite layer of graphene oxide and sulfonated polyether ether ketone the intermediate layer [6]. Montmorillonite nanoclay with a large surface area and containing alumina and silicate components was also demonstrated by Eswaraswamy et al. to be an efficient catalyst for water dissociation [7]. Celik et al. further explored that ferric chloride as an interface layer catalyst enhanced acid production and induced lower electrical resistance during bipolar membrane electrodialysis [8]. Nano-MoS_2_ was synthesized by Rathod et al. because of its similar conductivity to that of graphene and carbon nanotubes [9]. However, these bipolar membranes are only used for electrodialysis experiments under laboratory conditions, and there are many problems to be overcome for industrial applications. Problems include energy consumption, current efficiency, membrane lifetime and optimization of equipment parameters. Poor resolution of these issues can lead to higher production costs and is not conducive to mass production.

## 2. Application of Bipolar Membranes

**Figure 1:**
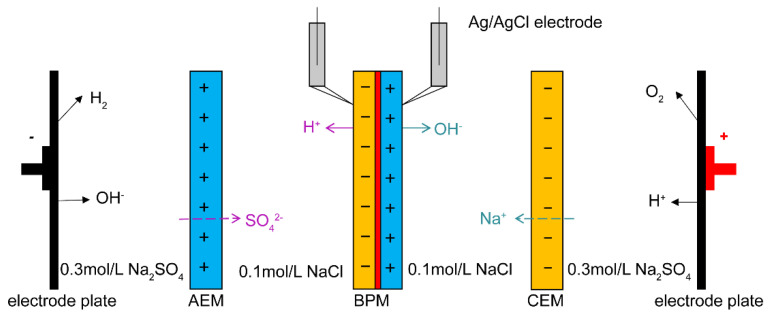
A bipolar membrane electrodialysis device and measurement principle of I-V curves.

Bipolar membrane electrodialysis (BMED) is a new technology that combines the separation function of electrodialysis with the water dissociation at the interface layer of bipolar membranes, which can convert salts into the corresponding acids and bases. Therefore, the two combinations are often used in industry to produce acids and bases. Figure 1 is an example of an electrodialysis device. BMED can be used for the clean production of organic acids, inorganic acid and bases, including succinic acid [10,11], salicylic acid [12,13], lactic acid [14,15], amino acids [16], hydrochloric acid [17], sulfuric acid [18,19], phosphoric acid [20] and choline hydroxide [21]. Sheldeshov et al. introduced ionic polymers with catalytically active phosphoric acid groups into bipolar membranes and studied the process of recovering nitric acid and sodium hydroxide from a sodium nitrate solution [22]. The use of bipolar membrane units for the production of nitric acid and sodium hydroxide containing phosphoric acid groups could achieve higher concentrations, current efficiencies, productivity and lower energy consumption and sodium nitrate contamination levels. Venugopal et al. functionalized and modified polysulfone (PSu) with resin and fiber reinforcement to prepare bipolar membranes containing Pt catalysts. The sodium chloride solution in brackish water with a concentration of 5~25 g/L was desalinated by electrodialysis desalination process. During the desalination treatment, the current efficiency was 82.5%, the energy consumption was 0.52 Wh, and the acid-base production was 0.006 mol/L acid and 0.006 mol/L base [23]. According to the unique hydrolysis mechanism and desalination effect of BMED, it could also be applied to the treatment of high-salt wastewater [24,25,26]. At the same time, bipolar membranes also play an irreplaceable role in the fields of the chemical industry, food processing, environmental protection, CO_2_ emission reduction, photocatalytic hydrogen production, biotechnology and the pharmaceutical industry [27,28,29,30,31,32,33].

The burning of fossil fuels and the convenient living of people produce large amounts of CO_2_. The CO_2_ contained in these waste gases cannot be directly utilized. Therefore, the combination of CO_2_ capture and BMED technology can realize the utilization and regeneration of CO_2_. CO_2_ is captured using potassium hydroxide and sodium hydroxide solutions to produce the corresponding carbonate solution or bicarbonate solution [34,35,36]. The bipolar membrane electrodialysis technology electrolyzes the salt solution to produce pure CO_2_ gas. This CO_2_ regeneration process significantly reduces energy costs and is environmentally friendly. CO_2_ capture can also be performed by passing the exhaust gas into organic reagents such as methionine salts [37] or aniline [38]. In addition, BMED technology can treat hazardous substances and recover other resources such as organic acids in industrial waste [39,40,41]. Noguchi et al. used multi-step BMED to concentrate a sodium borate solution with an initial concentration of 100 mg/L to a solution containing approximately 10,000 mg/L of boron [42]. In the discharged beverage industry, wastewater, a combination of concentration electrodialysis and BMED, Lameloise et al. successfully obtained L-malic acid recoveries of 93–97% [43].

## 3. Study of the Interface Layer Catalyst

The interfacial layer is the main place where water dissociation occurs in bipolar membranes. Assuming that this phenomenon was explained by the usual water dissociation mechanism, according to Simons’ theory [44], the calculated fluxes of H^+^ and OH^−^ were about 2 × 10^−9^ mol/m^2^ s, while the actual fluxes in bipolar membranes were as high as 10^−2^ mol/m^2^ s, the calculated value was inconsistent with the experimental value, indicating that the water dissociation of the bipolar membrane is not the same as the water dissociation in the normal state. Therefore, there is an effect of promoting water dissociation.

According to the studies of the water dissociation behavior of a bipolar membrane by many researchers, there are two main theoretical models to explain this phenomenon. The second Wien effect is a theoretical model based on the hydrolysis of weak electrolytes under high electric fields [45]. The theory holds that when the applied electric field is high enough, the conductivity of electrolyte increases rapidly with the electric field, causing Ohm’s law to fail. Applying the second Wien effect to bipolar membranes, water dissociation occurs in a sharp depletion region between the anion and cation exchange layers, where water is broken down into H^+^ and OH^−^ as a weak electrolyte. In Onsager’s experiments, the electric field strength in the depletion region was as high as 10^8^ V/m, and the water dissociation rate constant was 10^7^ times higher than when no applied voltage was applied. Surprisingly, the theory cannot continue to be implemented in the experiment for the electric field intensity higher than 10^8^ V/m. To investigate the cause, other effects that high electric fields may bring are ignored.

Since then, Simons had conducted numerous experiments to investigate functional groups and proposed that the hydrolysis of water molecules into H^+^ and OH^−^ generated a protonation and deprotonation reaction model by interacting with the membrane’s fixed charge groups [44,46], i.e., the chemical reaction theoretical model (CHR). According to the CHR hypothesis, water dissociation is primarily driven by active group catalysis, i.e., protonation and deprotonation of the active group. The application of this model to the water dissociation mechanism of the bipolar membrane interface layer showed that the water dissociation depends on the properties of charged groups, and not all functional groups made a significant contribution to water dissociation. Other studies had found that protonation and deprotonation reactions were not limited to membrane fixed charge groups [47], and the role between other active points in the membrane (such as metal oxides, heavy metal impurities, and metal complexes) and water molecules might also be applicable.

**Figure 2:**
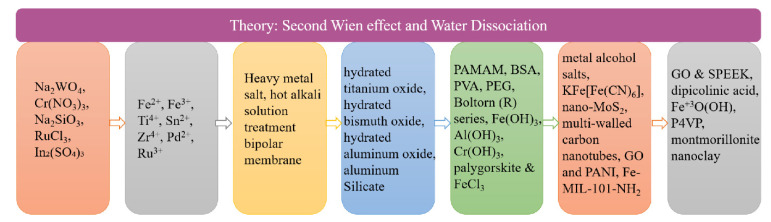
A schematic diagram of the development of intermediate layer materials. Polyamidoamine (PAMAM); bovine serum albumin (BSA); polyvinyl alcohol (PVA); polyethylene glycol (PEG); graphene oxide (GO); polyaniline (PANI); sulfonated polyether ether ketone (SPEEK); poly(4-vinylpyrrolidine) (P4VP).

**Figure 3:**
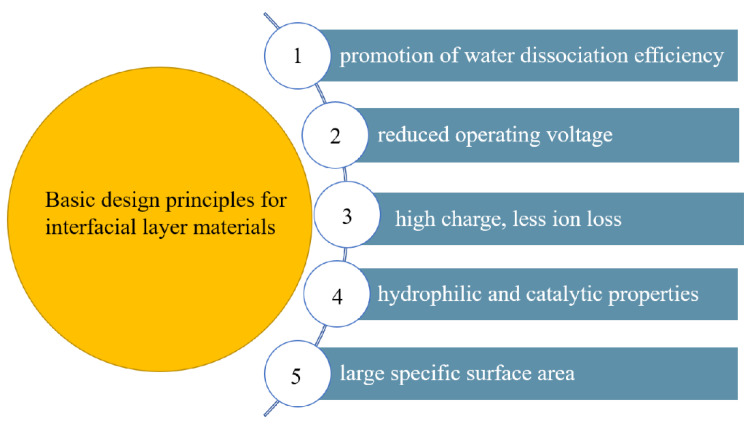
The basic principles for selecting interlayer materials.

The bipolar membranes currently used could not bear high temperatures with low mechanical strength and poor conductivity. New bipolar membranes which have good permeability, high permeaselectivity and good stability are urgently needed. Selectivity is an important parameter in the BMED process, if it is necessary to electrolyze ion solutions containing different valence states to produce a specific substance. Bipolar membranes with high selectivity improve the purity of the product. On the contrary, it not only reduces the product purity but also increases the energy consumption in the electrodialysis process [48]. Since water molecules in the interface layer are split to generate an acid-base solution, it is further required that the bipolar membranes need to have great resistance ability for acids and bases. The methods to improve the performance of bipolar membranes are mainly divided into the modification of the anion and cation exchange layers and the introduction of catalysts in the interface layer. Figure 2 depicts the development process of the interfacial layer materials. The transmembrane voltage of bipolar membranes is an important factor affecting the performance of bipolar membranes, which mainly depends on the voltage drop of the interface layer. Catalysts are added to the interface layer to promote the water dissociation, reduce the activation energy of the water dissociation reaction, increase the water dissociation rate, reduce the membrane impedance, and make the BPM process more energy efficient. The basic principles for the selection of the interfacial layer materials are included in Figure 3.

Previous research found that inorganic electrolytes with amphoteric properties [44] (such as sodium metasilicate, chromium nitrate, ruthenium trichloride, or indium sulfate) and heavy metal ions (Fe^2+^, Fe^3+^, and Ti^4+^, etc.) on CEL/AEL catalyzed interface layer water dissociation and reduced bipolar membrane voltage. Moreover, weak acids, amino functional groups, carboxylic acids and pyridines had catalytic effects on the interface layer of the bipolar membrane, reducing the voltage of the bipolar membrane [49]. Although the expected results were obtained, these results did not fully describe the precise interface layer water dissociation process, and more research is still needed to properly understand the water dissociation behavior as well as the wider catalytic components.

The research on bipolar membranes mainly focuses on introducing suitable catalysts to improve the water dissociation rate. Catalysts that have been used could be classified as organic and inorganic materials. Newly designed materials with multiple components as catalytic intermediate layers are being studied more frequently.

### 3.1. Organic Materials Act as Catalysts

Now that inorganic electrolytes can lower voltage drop, it is assumed that some organic macromolecules can do it as well. In the foreseeable future, organic macromolecules with hydrophilic, acidic, or basic functional groups have been discovered to lower transmembrane voltage and enhance bipolar membrane performance. Hydrophilicity, concentration effect and steric resistance effect were found to be the three main factors affecting the rate of bipolar membrane hydrolysis. The hydrophilicity of the catalyst increases the hydrophilic point of the interface region. Both the concentration effect and the potential resistance effect are related to the catalyst concentration. The increase of catalyst concentration increases the bipolar membrane water dissociation efficiency, which shows the concentration effect; conversely, it shows the steric resistance effect. The steric effect is also reflected in the structure of the catalyst itself. In the above discussion, the interfacial region is the main location where water dissociation occurs, which requires that the suitable interfacial layer thickness needs to be optimized. Too high a catalyst concentration increases the thickness of the interface layer and the bipolar membrane; therefore, an optimal concentration exists to make the bipolar membrane the most efficient for water dissociation.

**Figure 4:**
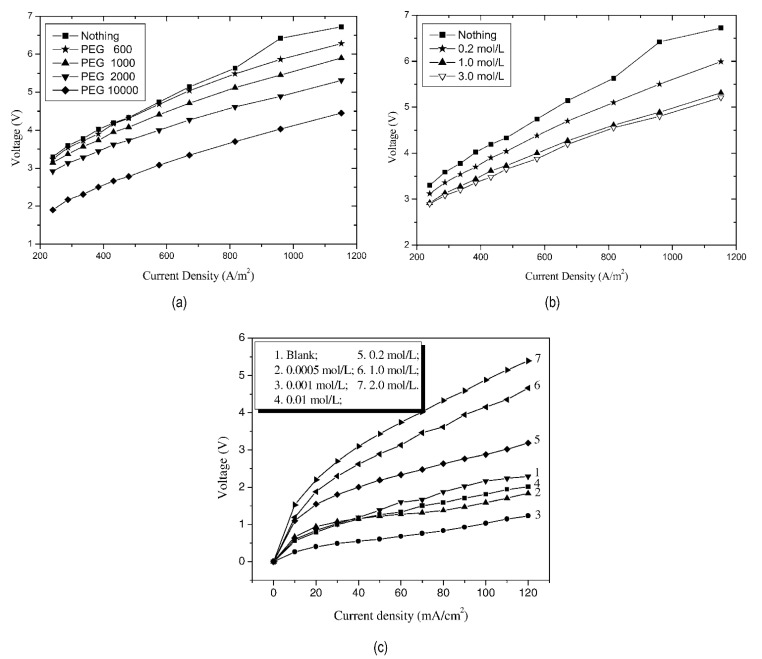
The I-V curves of bipolar membranes of which the anion exchange membranes have been immersed in (**a**) 1.0 M solutions of different weight PEGs and (**b**) different concentration solutions of PEG 2000. Reproduced with permission from Ref. [52], Copyright 2003 Elsevier Inc. (**c**) Relationship between current density-voltage and different PVA concentrations. Reproduced with permission from Ref. [53], Copyright 2004 Elsevier Inc.

Polyvinyl alcohol (PVA) and polyethylene glycol (PEG) were the commonly used organic catalysts and they had similar effects on bipolar membrane water dissociation [50,51,52]. With the adsorption of PVA and PEG to the interfacial layer, the water dissociation effect of the bipolar membrane promoted, and the water dissociation voltage reduced. A protonation–deprotonation reaction occurred between the hydrophilic hydroxyl functional groups and the water molecules in the BPM interface layer, and the number of hydrophilic sites in the interfacial region increased. The molecular weight of PEG was also a factor affecting the water dissociation of bipolar membranes, showing a positive correlation (in Figure 4a). However, PVA and PEG showed different effects at different concentrations (see Figure 4b,c). The high concentration of PVA inhibited water dissociation while the high concentration of PEG catalyzed water dissociation, because the thickness of the interfacial layer was excessively extended by PVA, which itself was due to the easy adsorption and accumulation of PVA on the membrane surface, as well as the strong interaction between PVA molecules [53].

**Figure 5:**
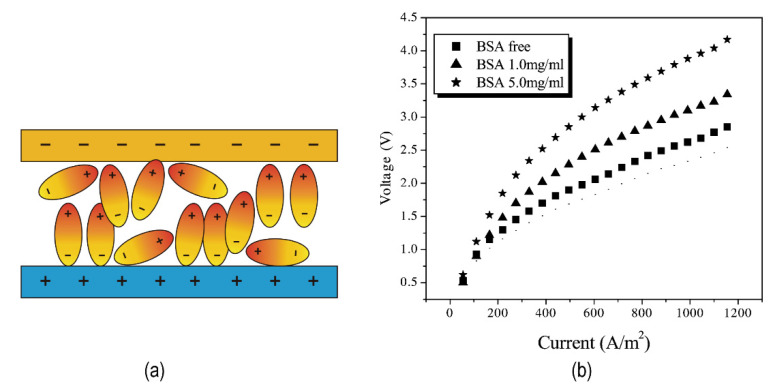
(**a**) The scheme of BSA adsorption (aggregation) in the junction; (**b**) Effect of BSA concentration in bipolar membranes on I−V curves. Reproduced with permission from Ref. [54], Copyright 2004 Elsevier Inc.

**Figure 6:**
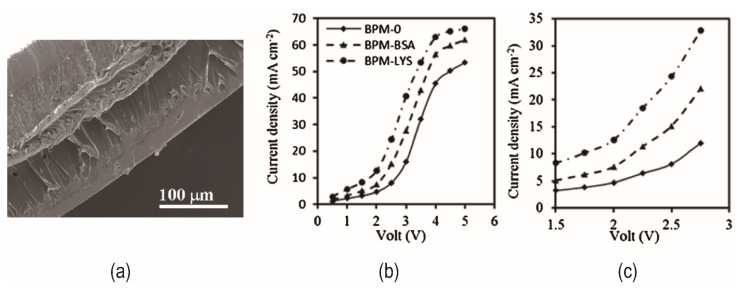
(**a**) SEM image of LYS as interfacial layer of bipolar membrane; (**b**) Current–voltage curves for different BPMs in equilibration with 0.10 M NaCl solution; (**c**) Partial magnification of (**b**). Reproduced with permission from Ref. [55], Copyright 2017 Elsevier.

The amphoteric substance bovine serum albumin (BSA) contains amino (weak acid) and carboxyl (weak base) groups but unexpectedly inhibited hydrolysis dissociation. Figure 5a shows a schematic diagram of the adsorption of BSA in the intermediate layer. Increased BSA concentration harmed the voltage drop of the membrane (illustrated in Figure 5b). The inhibition, according to Fu et al., came from the properties of the BSA molecule itself: the steric effect caused the interfacial layer to thicken, electrostatic interaction weakened the interfacial layer’s electric field, and the BSA made the interfacial layer more water-repellent [54]. These all play a negative role in water dissociation. While containing amino and carboxyl groups that promote hydrolysis does not necessarily exhibit the promotion of hydrolysis, the presence of other effects dominates the competition over catalytic effects. Compared with BSA, bipolar membranes containing lysozyme (LYS) possessed higher water dissociation efficiency and lower salt leakage values. The SEM image of the bipolar membrane prepared with LYS is shown in Figure 6a. I–V curves showed that LYS as an interface layer had a lower water dissociation voltage than BSA (in Figure 6b,c). LYS with acid and base groups is hydrophilic, which is beneficial to H^+^/OH^−^ transport [55].

**Figure 7:**
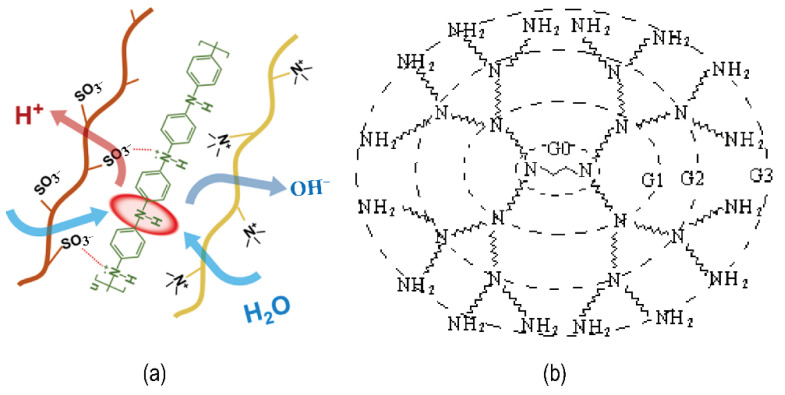
(**a**) A schematic diagram of water dissociation with PANI as catalysts; Reproduced with permission from Ref. [56], Copyright 2021 Elsevier B.V.; (**b**) Ideal molecular formula of PAMAM G3. Reproduced with permission from Ref. [58], Copyright 2004 Elsevier B.V.

Proton transfer processes are accelerated by the weakly basic amino functional group, which catalyzes hydrolysis. Chen et al. and Li et al. collaborated this concept by studying polyaniline (PANI) with a secondary amine group and polyacrylamide (PAM) with a primary amine group [56,57]. Figure 7a shows the schematic diagram of the PANI catalyzed hydrolysis. Similarly, starburst dendrimer polyamidoamine (PAMAM), a dendrimer with a high density of amine groups (see Figure 7b), could also be employed to aid bipolar membrane hydrolysis. Concentration and generation which affects the number of shells of the PAMAM molecules and in turn affects the number of amino groups are key variables to influence the water dissociation rate. The reason for this is the competition and compromise between the catalytic effect of the amine group and the steric effect of the macromolecule [58].

**Figure 8:**
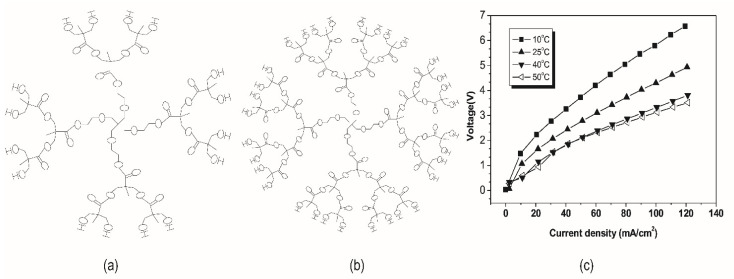
Ideal molecular formula of (**a**) Boltorn^®^ H20; (**b**) Boltorn^®^ H30; (**c**) The effect of temperature on the I–V curves of bipolar membranes modified by a 0.1 mg/mL Boltorn^®^ H20 aqueous solution. Reproduced with permission from Ref. [59], Copyright 2007 Elsevier Inc.

**Figure 9:**
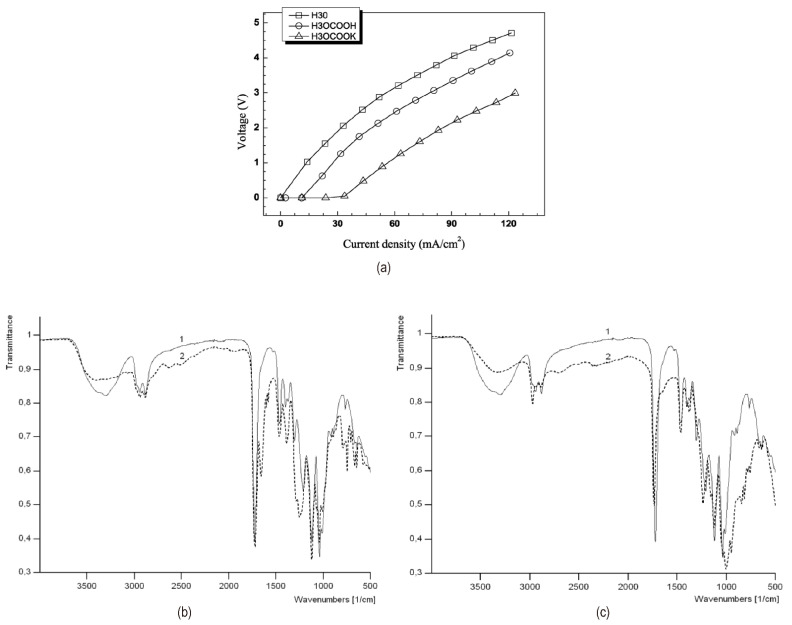
(**a**) The I–V curves of the bipolar membranes treated with nothing, 1.0 mg/mL H30-COOH, and 1.0 mg/mL H30-COOK at 25 °C. Reproduced with permission from [60], Copyright 2009 Elsevier B.V. (**b**) FT-IR spectra of H20 (1), and H20-COOH (2). (**c**) FT-IR spectra of H20 (1), and H20-PO_3_H_2_ (2). Reproduced with permission from Ref. [61], Copyright 2015 Elsevier B.V.

Like PAMAM, hyperbranched aliphatic polyester solutions (Boltorn^®^ series) with abundant hydroxyl groups (see Figure 8a,b) as end groups are expected to be effective in catalyzing the water dissociation in bipolar membranes. Its porous and loose structure makes it easy for water molecules to enter the middle layer of bipolar membranes, which plays a positive role [59]. Besides concentration and generation which affects the number of terminal hydroxyl groups in hyperbranched aliphatic polyester molecules, temperature impacted the degree of water dissociation (shown in Figure 8c). An optimal temperature gave the lowest voltage across the membrane after a fixed concentration. Intermolecular hydrogen bonds between water and hyperbranched polyester molecules at low temperatures were weak. With the temperature increasing, the stable intramolecular hydrogen bonds were broken, and it was easier to form intermolecular hydrogen bonds, thereby catalyzing the splitting of water. Xue et al. [60] and Zabolotsky et al. [61] carboxylated and phosphorylated Boltorn^®^, respectively, and the enhanced hyperbranched aliphatic polyesters further increased the rate of hydrolysis (Figure 9a). Figure 9b,c showed the FT-IR spectra of carboxylated and phosphorylated H20. The -PO_3_^2−^ group has higher catalytic activity in promoting the hydrolysis reaction because it has better hydrophilicity, strong spatial effect and better ability to form intermolecular hydrogen bonds with water molecules.

In general, weak acids such as carboxylic acid and phosphoric acid as well as weak bases such as pyridine and amino group are good catalysts to reduce water dissociation. Hydroxy groups that readily form intermolecular hydrogen bonds with water molecules could also accelerate water dissociation. However, BSA is a particular example, and although BSA contains amino and carboxyl groups, it suppresses water dissociation.

### 3.2. Inorganic Material Acts as a Catalyst

**Figure 10:**
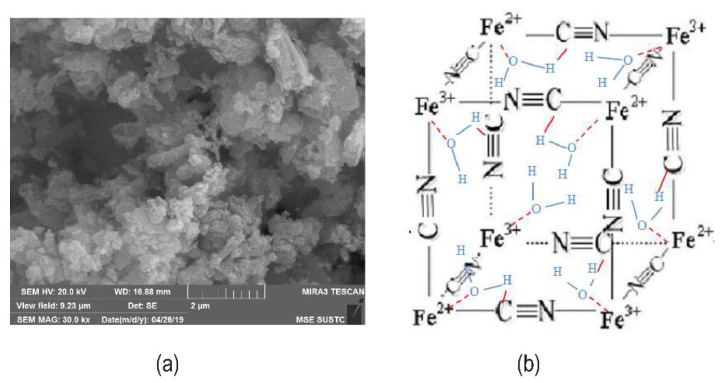
(**a**) SEM image of KFe[Fe(CN)_6_] deposited on CEL surface; (**b**) Mechanism of water dissociation in KFe[Fe(CN)_6_] cubic crystal. Reproduced with permission from Ref. [62], Copyright 2019 Elsevier B.V.

Currently, inorganic catalysts containing metal elements are the main trend of research, such as metal oxides/hydroxides (Fe(OH)_3_, Al(OH)_3_), and metal ion complexes. The dissociation of water on metal oxides and hydroxides occurs by adsorption of water onto the surface followed by transfer of protons from the water to neighboring oxygen atoms, resulting in hydroxide ions. However, in the actual electrolysis process, the catalyst containing metal ions has the disadvantage that the metal ions are easily lost due to their small size, and an effective method is to immobilize the metal ions. Rational utilization of metal oxide materials in terms of metal oxide surface species and utilization efficiency helps to identify the effect of catalytic activity species and content on the rate of water dissociation during water dissociation. Inorganic catalysts could reduce the polarization effect between sulfonic acid and quaternary ammonium at high concentrations. In the case of metal ion complexes (such as KFe[Fe(CN)_6_] [62]), the intermediate layer’s strong catalytic activity could primarily offer multiple catalytic sites (see Figure 10b) to draw water from the ion exchange layer to the space charge area and become more hydrophilic, therefore speeding up the proton transfer process. Figure 10a shows the SEM image of the deposited KFe[Fe(CN)_6_].

**Figure 11:**
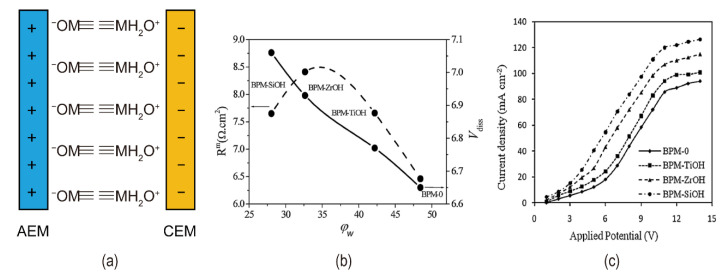
(**a**) A schematic diagram of ≡M−OH as a bipolar membrane catalyst; (**b**) Variation of the R_m_ and V_diss_ with the water content for different BPMs with different metal alkoxide catalysts in equilibration with a NaCl solution (0.5 M); (**c**) Current–voltage (I−V) curves for BPMs with different metal alkoxide catalysts in equilibration with a 2.0 M NaCl solution. Reproduced with permission from Ref. [66], Copyright 2012 Elsevier Ltd.

Jeevananda et al. immersed cation exchange membranes in ferric chloride and sodium hydroxide solutions successively, causing the iron(III) hydroxide to be immobilized on the membranes. The metal content affected the catalytic reaction of the interface layer, and the current efficiency of the BPM containing 5% (*w*/*v*) Fe(III) hydroxide exceeded 95% [63]. While Kang et al. used electrodeposition [64,65] to immobilize Fe(III) hydroxide more efficiently than the method used by Jeevananda et al. The bipolar membrane hydrolysis rate and water content were greatly enhanced when Rajesh et al. employed thin membranes of various metal alcohol salts (M(OR)_n_, M for Si, Ti, or Zr, R for -(CH_2_)_n_-CH_3_) as the interfacial layer [66] (see Figure 11a). Metal alkanes have hydration capabilities, and they operate as active surface sites (M-OH) in the ion-exchange membrane matrix, enhancing the transfer of protons from water molecules to fixed charge groups. BPM-SiOH has a faster rate of hydrolysis due to the presence of free hydroxyl or silanol groups in the membrane (illustrated in Figure 11b,c).

**Figure 12:**
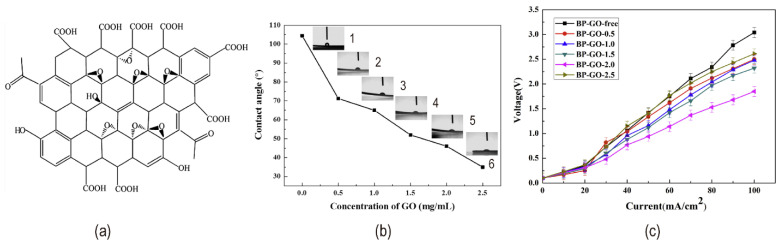
(**a**) A schematic diagram of the molecular structure of graphene oxide; (**b**) Contact angle tests with different GO concentration solution: (1) 0 mg/mL; (2) 0.5 mg/mL; (3) 1.0 mg/mL; (4) 1.5 mg/mL; (5) 2.0 mg/mL; (6) 2.5 mg/mL. (Spraying different concentration of GO two times on anion membrane); (**c**) I–V curves of bipolar membranes with different concentrations of GO solutions. Reproduced with permission from Ref. [70], Copyright 2017 Elsevier B.V.

**Figure 13:**
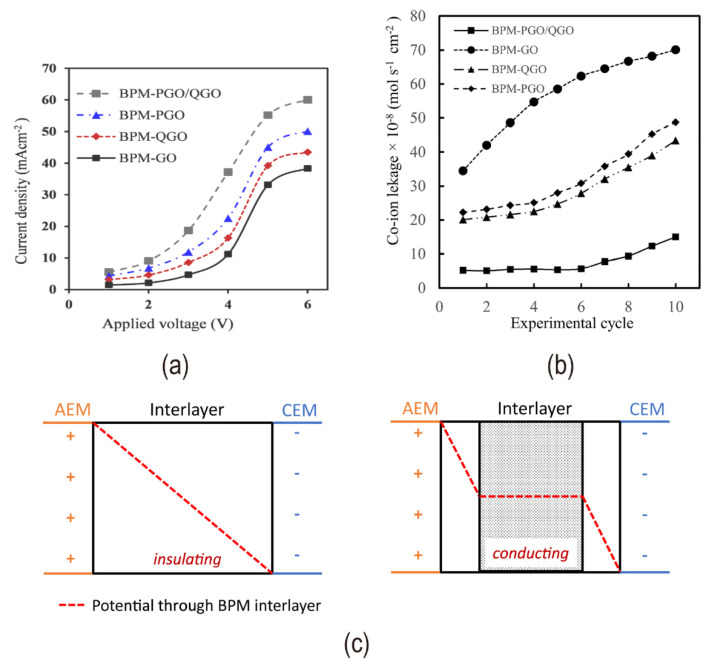
(**a**) I−V curves of graphene oxide and functionalized graphene oxide as the interface layer of bipolar membranes; (**b**) Co−ion leakage for different BPMs after different experimental cycles. Reprinted (adapted) with permission from Ref. [71], Copyright 2018 American Chemical Society; (**c**) Schematic drawing of the potential through electronically insulating and electronically conducting interface layer materials. Reproduced with permission from Ref. [72], Copyright 2019 Springer Nature.

Due to the planar nature of the BPM interface layer, two-dimensional materials such as graphene oxide (GO) could be utilized to improve catalytic performance. The molecular structure of GO is shown in Figure 12a. Through oxygen-containing groups (such as hydroxyl, carboxyl, carbonyl, and epoxide) and hydrophilicity, GO enhances the hydrophilic sites on the interfacial layer [67]. Figure 12b illustrates the increasing hydrophilicity with the increase of GO concentration. As a result, forming hydrogen bonds and polar interactions with water is simple, promoting water dissociation [1,55,68]. Carboxylic acid groups have the highest reactivity for water dissociation among them. Because graphene oxide has a high number of active sites per unit volume, it has a high catalytic activity, therefore using thin layers allows for higher electric field strength and hence a faster rate of water splitting [69,70]. Therefore, the second Wien effect and the chemical reaction model are well balanced. The amount of GO loaded has a significant impact on the hydrolysis voltage, as evidenced by two factors: increased catalyst loading increased the number of catalytic sites; excessive loading increased the interface layer thickness, lowering the electric field intensity (illustrated in Figure 12c). The mixture of phosphorylated graphene oxide (PGO) and quaternized graphene oxide (QGO) which were GO functionalized as the interface layer significantly reduced the resistance and improved the stability of BPM. A dual charged PGO/QGO interface layer, which is both acidic and basic, provided a hydrophilic bipolar environment and promoted the polarization of water molecules [71] (see in Figure 13a,b).

The conductivity of the catalyst, in addition to hydrophilicity and catalytic activity, influences the hydrolysis of bipolar membranes. Water splitting was catalyzed by the conductive interface layer material, even though it lacks catalytic active sites. The reason for this is that the interface layer’s electronic conductance might boost the strength of the electric field in the mobile ions’ depletion zone [72] (in Figure 13c). The performance of bipolar membranes with graphene and carbon nanotubes as interface layers is independent of the interface layer thickness but lower than that of graphene oxide.

Li et al. explored the effect of bipolar membranes with molybdenum disulfide (MoS_2_) as the interface layer on water dissociation [73]. The I–V curves showed that the voltage drop of the bipolar membrane with the MoS_2_ interface layer was reduced compared with that of the bipolar membrane without MoS_2_. This illustrated that MoS_2_ was a good catalyst to catalyze the splitting of water. MoS_2_ is a hydrophilic substance, and the water dissociation reaction mechanism of the interface layer needs further investigation.

### 3.3. Newly Designed Materials with Multiple Components

If metal ions are used as catalytic interface layer, they are prone to ion leakage due to their small size. Therefore, after a period of use, the current efficiency decreases and the voltage drop of the membrane increases, which is not conducive to the continued use of the membrane. Such bipolar membranes have a short lifespan. Mixing macromolecular substances with metal ions as catalysts could fix the metal ions and improve the service life of the membrane. The synergistic effect of mixing two components could be considered to improve the shortcomings of single species for catalytic water dissociation.

**Figure 14:**
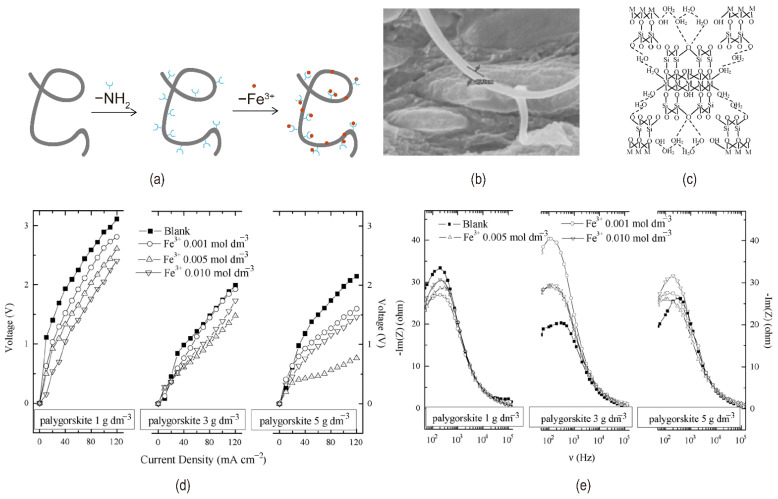
(**a**) The process of loading Fe^3+^ on multi−walled carbon nanotubes; (**b**) SEM image of multi−walled carbon nanotubes loaded with Fe^3+^. Reproduced with permission from Ref. [74], Copyright 2017 Elsevier B.V.; (**c**) Chemical structure of palygorskite. M stands for Al^3+^ or Mg^2+^. Effects of Fe^3+^ and palygorskite concentrations on (**d**) I−V and (**e**) EIS curves of bipolar membranes. Reproduced with permission from Ref. [75], Copyright 2008 Elsevier B.V.

**Figure 15:**
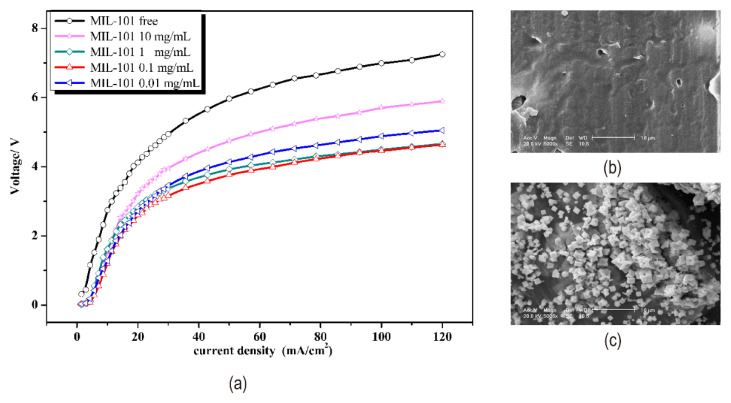
(**a**) I–V curves of different concentrations of Fe-MIL-101-NH_2_ as the interface layer of bipolar membranes. Reproduced with permission from Ref. [77], Copyright 2016 Elsevier B.V. SEM images of the AEL surface (**b**) before and (**c**) after treatment with 5000 ppm silver nitrate aqueous solution. Reproduced with permission from Ref. [79], Copyright 2005 Elsevier Inc.

For example, acid-oxidized multi-walled carbon nanotubes (MWCNTs) or palygorskite were added to Fe^3+^-containing bipolar membranes. Figure 14a shows the process of loading Fe^3+^ on multi-walled carbon nanotubes. SEM image of multi-walled carbon nanotubes loaded with Fe^3+^ was shown in Figure 14b. Multi-walled carbon nanotubes have a high specific surface area and many unsaturated bonds that form hydrogen bonds with water, leading to increased hydrophilicity. Acid oxidation-treated multi-walled carbon nanotubes contain -COO^−^ groups, and Fe^3+^ was immobilized by binding to the -COO^−^ groups. Palygorskite help split water with rich hydroxyl and silica groups [74]. The molecular structure of palygorskite is shown in Figure 14c. Simultaneously, palygorskite shields Fe^3+^ from considerable loss and stabilizes the membrane’s water splitting properties [75,76] (illustrated in Figure 14d,e). For coordination between metal ions and organic ligands, a novel catalytic metal-organic framework (Fe-MIL-101-NH_2_) existed, which can reduce metal ion loss. In addition, Fe-MIL-101-NH_2_ with a porous shape speeded up water splitting in bipolar membranes (see Figure 15a) since it includes both weak acid and metal groups [77]. Fu et al. previously investigated that PAMAM could modify the interface layer of bipolar membranes. Since polyamide amines can form complexes with some heavy metal ions, the effect of Cr(III) incorporation into the interface layer containing PAMAM continued to be investigated. Cr(III) increased the catalytic site of the complex and PAMAM immobilized the Cr(III) ion in the intermediate layer, resulting in a mixture of PAMAM and Cr(III) with higher efficiency of water dissociation [78]. It has been demonstrated that silver chloride is an intermediate layer catalyst to accelerate water dissociation. And the mixture of silver chloride and gelatin as an intermediate layer water cleavage reaction was faster and the voltage across the membrane was smaller. The SEM images of the AEL before and after loading silver chloride are shown in Figure 15b,c. Hydrophilic gelatin reduces the potential at low concentrations. Gelatin is a protein, so high concentrations led to higher voltage drops due to spatial effects and electrostatic interactions [79].

Graphene oxide is an efficient catalyst for water dissociation, but concentration effects prevent the concentration of GO from being too high. The incorporation of high surface area organic conductor polyaniline (PANI) to form GO-PANI nanocomposites provided synergistic effects to enhance the conductivity of PANI and mitigate GO aggregation [80]. Moreover, with the strong hydrophilicity of GO and the small space effect caused by its ultrathin property, there was a synergistic catalytic hydrolysis reaction between GO and PVA to achieve a lower voltage drop [81].

There are few recent studies on organic materials as interface layer catalysts [56,82,83], more research is mainly focused on composite materials and inorganic materials, such as iridium dioxide [84], graphene oxide [69,85], ruthenium [86], silver [87], chromium hydroxide [88], iron hydroxide [88,89,90], aluminum hydroxide [72,91,92], iron chloride [8], Fe^3+^O(OH) [93] and etc. A more in-depth study of graphene oxide has been carried out to mix it with organic materials as catalysts to improve water dissociation performance [6,80,81].

## 4. Effect of the Interfacial Structure

Besides interfacial catalysts, rational design of interfacial structures is also a significant approach to improving the performance of bipolar membranes. In the actual operation process, various situations are not conducive to electrolysis. If the two membrane layers are loosely bound, the membrane layers will separate at lower current densities, which is known as ballooning. This phenomenon is caused by the osmotic pressure generated by the undepleted ions in the interfacial region at low or no current. The alkaline solution generated by the BMED process easily absorbs CO_2_ in the air to generate HCO^3−^ and there is a reversible balance between CO_2_ and HCO^3−^. The high concentration of HCO^3−^ near the interface region makes CO_2_ stay in the membrane, resulting in delamination. This is another reason for delamination or ballooning. Thus, the importance of interfacial structures can be seen to the stability of BPMs.

**Figure 16:**
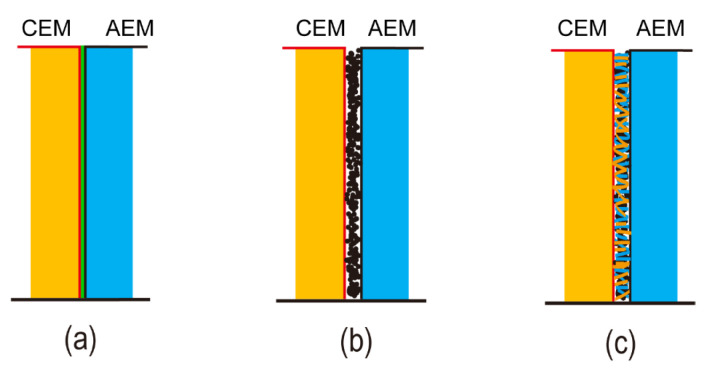
The anion and cation exchange layers and the interface layer contact to form different junction structures: (**a**) smooth structure; (**b**) 2D structure with catalyst; (**c**) 3D structures containing catalysts prepared by electrospinning and other techniques. Reprinted (adapted) with permission from Ref. [94], Copyright 2021 American Chemical Society.

The interfacial regions are mainly classified into three types depending on the type and structure of the interfacial catalyst as well as the membrane layers themselves and how they are in contact with each other [5,94] (see Figure 16). Due to the preparation technology, the intermediate layer and the ion exchange layer penetrate each other, and are impermeable to the other ion exchange layer, forming a smooth and sharp two-layer structure. On the contrary, the intermediate layer and the two ion exchange layers are impermeable to each other, forming a three-layer structure (Figure 16a). A rough surface of CEL/AEL polished by a tool similar to sandpaper combines with the intermediate layer to form a wrinkled interface region. This treatment method greatly increases the contact area. The interfacial layers of many bipolar membranes are generally heterogeneous. Each monopolar layer of heterogeneous bipolar membranes (HBM) was obtained by dispersing resin powder in an inert polymer solution (binder) and hot pressing/casting layer by layer. The presence of ion exchange resin in the monopolar membrane layer increases the surface roughness of the HBM and then increases the contact area with the intermediate layer [6].

According to different preparation methods, the above several different interface structures are generally obtained. A smooth interfacial structure could be described by a one-dimensional (1D) or 2D mathematical model and this is usually used in bipolar membranes (Figure 16a). Smooth interface layers could be composited by commercial anion and cation exchange layers with smooth surfaces. Certainly, casting the polymer solution onto a commercial ion exchange membrane or another preformed exchange layer could also form a smooth intermediate layer. Casting a second membrane from the solution rather than the pre-prepared membrane exhibited close contact and better performance of BPM. In some cases, the surface of roughened AEL/CEL promotes tight bonding of the two layers and increases contact area. To some extent, this reduces the overpotential required to drive water dissociation and ion separation. Using a catalyst similar to graphene as the interface layer of the bipolar membrane, the interface region presents a 2D structure (see Figure 16b).

To prepare bipolar membranes with excellent performance, it is necessary not only to select the best components but also to use techniques that could form membrane layers and obtain ideal interfacial structures. In addition, introducing ion-exchange groups and catalytic functions into the selected materials is essential in the process of synthesizing the bipolar membrane. For the anion and cation exchange layers, the degree of functionalization i.e., the charge density and the thickness of the monopolar layer affect the initial water dissociation potential (U_diss_) of the bipolar membrane. U_diss_ is reduced by increasing charge density and decreasing the thickness of the monopolar layer. Thinner layers, on the other hand, increase the chance of ion crossover. As a result, the layer thicknesses, along with the interface layer, must be carefully optimized.

**Figure 17:**
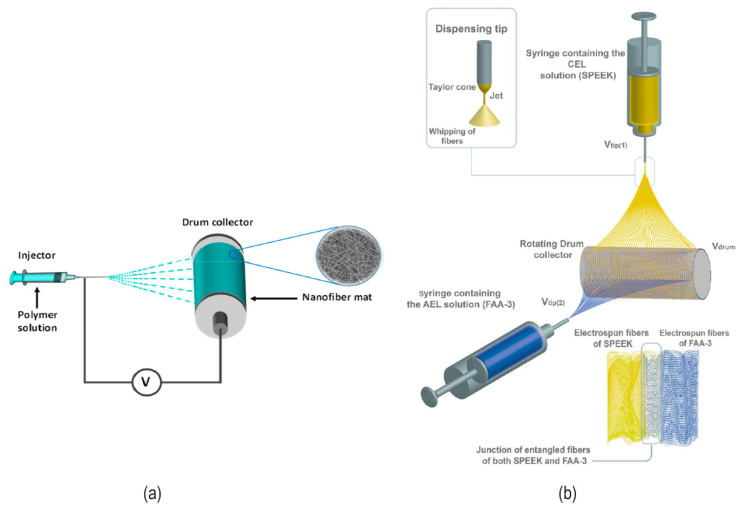
(**a**) The operating principle of electrospinning technology. Reprinted (adapted) with permission from Ref. [94], Copyright 2021 American Chemical Society. (**b**) Dual electrospinning process of anion and cation exchange fibers. Reprinted (adapted) with permission from Ref. [83], Copyright 2021 American Chemical Society.

**Figure 18:**
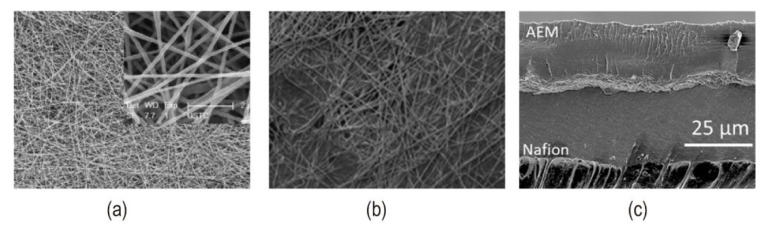
(**a**) SEM image and fiber diameter distribution of PEG prepared by electrospinning. Reproduced with permission from Ref. [95], Copyright 2016 Elsevier B.V. (**b**) SEM image of co-electrospun junction with GO. (**c**) SEM image of the cross-section of 3D BPM with GO as the interface layer catalyst. Reprinted (adapted) with permission from Ref. [85], Copyright 2020 American Chemical Society.

The macro roughened membrane and ion exchange particles introduced at the interface improved the contact area of the interface to some extent in early study. Unfortunately, the membranes with opposite charges cannot be completely in touch, there remain residual pores, and the interface composite layer’s ionic conductivity is low. Hot pressing, adhering, casting, and chemical modification processes are the most common ways of preparing bipolar membrane technology. The bipolar membrane interfacial region obtained by these conventional methods is a 2D junction structure (Figure 16a). These approaches can cause mutual penetration and delamination between the AEL and CEL, resulting in the interface layer forming a high resistance region and increasing the bipolar membrane’s working voltage. The thickness of the intermediate layer can be precisely controlled using various modern preparation technologies. An ultra-thin nano intermediate layer can be created using the spraying method [70]. Electrospinning is a technology that has gotten considerable attention in recent years. It also offers a unique method of controlling the thickness of the interfacial layer and overcoming the limitations of lamination and casting procedures. Electrospinning technology sprays anion-exchange and cation-exchange polymer solutions under the action of high electrostatic force, the solvent is evaporated and finally, a nanofiber interface structure is formed (Figure 17a). Compared with conventional 2D junction bipolar membranes, this 3D junction (Figure 16c) has a higher interfacial area where the catalyst can be fully covered. At the same time, lower transmembrane voltage drop, and more stable membrane performance were exhibited at high current density. Pan et al. used electrospinning to create an interface layer containing PEG in addition to the anion and cation exchange layers. By controlling the electrospinning time, the optimized bipolar membrane has a superior transmembrane voltage of 2.5 V at 100 mA/cm^2^ [95]. Many researchers constructed a novel 3D junction with interpenetrating anion and cation exchange membrane polymer fibers by double nanofiber electrospinning with a significant increase in catalytic sites (Figure 17b). Shen et al. added Al(OH)_3_ catalyst nanoparticles to the 3D interface layer and observed that at current densities up to 1.1 A/cm^2^, there is no delamination in 3D bipolar membranes, and U_diss_ could be as low as about 0.75 V [92]. Hohenadel et al. explained some hydrocarbon-based polymers had poor adhesion due to their relatively high glass transition temperature, which limited the formation of a strong interface layer [91]. Chen et al. coupled graphene oxide, one of the most promising water dissociation catalysts in recent years, with electrospinning for the first time to construct bipolar membranes. They found that the hot-pressed GO-containing 2D BPM worked similarly to the commercial Fumasep BPM at a current density of 500 mA/cm^2^ [85]. 3D BPM had lower resistance, higher hydrolysis efficiency, and more stability at high current density. The addition of the organic polymer poly(4-vinylpyrrolidine) (P4VP) to the 3D junction also confirmed an enhancement of the bipolar membrane water dissociation rate [83]. Figure 18a–c showed the SEM images of the bipolar membranes synthesized using electrospinning.

In a nutshell, electrospinning technology is easy to use. This approach overcomes the constraints of bipolar membrane’s 2D structure, expands it to a 3D structure, improves bipolar membrane performance, gives a new idea for bipolar membrane preparation, and represents a new development direction in bipolar membranes preparation [95,96,97].

**Figure 19:**
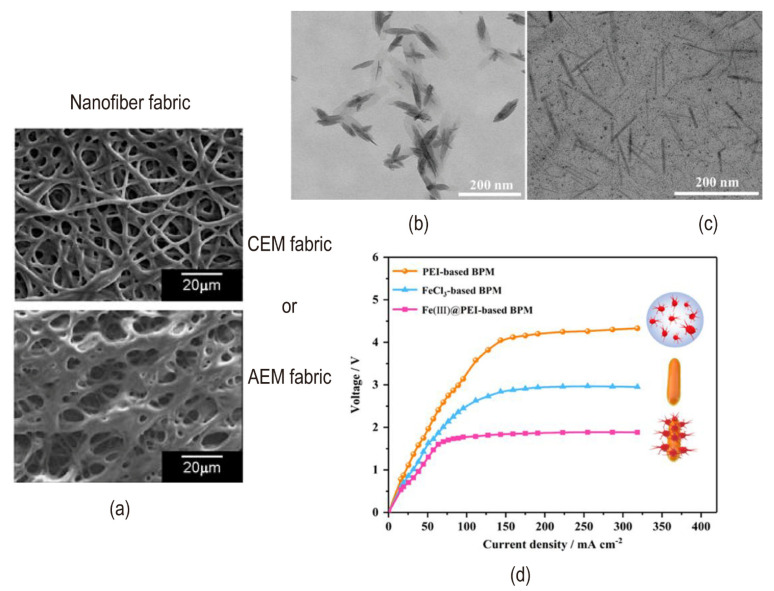
(**a**) SEM image of ion-exchanged nanofiber fabric as an intermediate layer. Reproduced with permission from Ref. [98], Copyright 2006 Elsevier Inc. The TEM images of (**b**) FeCl_3_ and (**c**) Fe(III)@PEI as the catalytic interface layer. (**d**) I–V curves of bipolar membranes with different catalysts of PEI, FeCl_3_ and Fe(III)@PEI. Reprinted (adapted) with permission from Ref. [99], Copyright 2020 American Chemical Society.

One technique to improve the specific surface area of catalysts is to prepare them into fibrous shapes. Wakamatsu et al. used electrospray deposition (ESD) to create a higher specific surface area (BET surface area of 600 m^2^/g) anion exchange fibers (AEF) compared to cation-exchange fibers, which aid to improve the contact surface area between AEF and CEL in addition to the electrostatic spinning approach [98] (Figure 19a). The synergy of terpyridine and quaternary pyridine groups contained in AEF fabrics with the high specific surface area of the proton deprotonation reaction enhances the water dissociation in the intermediate region of the BPM.

Aside from the preparation process, the catalysts’ structure is also important for hydrolysis. The structure of the recently studied Fe complex catalyst is important for hydrolysis. For example, the cubic structure of KFe[Fe(CN)_6_] fixed on the cation exchange layer induced a positive effect on water dissociation behavior. The crystal structure containing multiple positively charged Fe^2+^ and Fe^3+^ and negatively charged CN^−^ greatly increased the possibility of binding with water molecules, which more catalytic sites increased the occurrence of water dissociation behavior [62]. Complex solutions can be prepared either by reacting to two inorganic materials or by thoroughly blending inorganic and organic materials. In the previous discussion, inorganic compounds containing metal ions suffered from the disadvantage of metal ion loss due to the prolongation of electrodialysis time and the operation at a high current density. Through the coordinated interaction of amino groups in PEI to Fe(III) centers (Fe(III)@PEI), the nano-structure complex composed of polyethyleneimine (PEI) and FeCl_3_ enhanced the stability of bipolar membranes. The TEM images of FeCl_3_ and Fe(III)@PEI as the catalytic interface layer were shown in Figure 19b,c. Figure 19d illustrated the I–V curves of bipolar membranes with different catalysts of PEI, FeCl_3_ and Fe(III)@PEI. It showed that Fe(III)@PEI had a better hydrolysis effect. The synergy mechanism is studied that Fe(III)@PEI complexes formed a smaller size compared to FeCl_3_, which provided more active sites for water dissociation in the interface layer [99]. Meanwhile, the binding ability among the three membrane layers was possibly enhanced by electrostatic interaction or chemical reaction between amino groups in Fe(III)@PEI complex solution with brominated benzyl groups in the AEL and carboxyl groups in the CEL. In summary, an extremely important factor to be considered is the structure of the interface layer and it determines the water dissociation capacity of the bipolar membrane. However, studies on materials like Fe(III) (a metal ion) and amino groups (a weak base) provide more active sites that are unsystematic. Further discussion on the effect of complex catalysts on the water dissociation mechanism is required.

**Figure 20:**
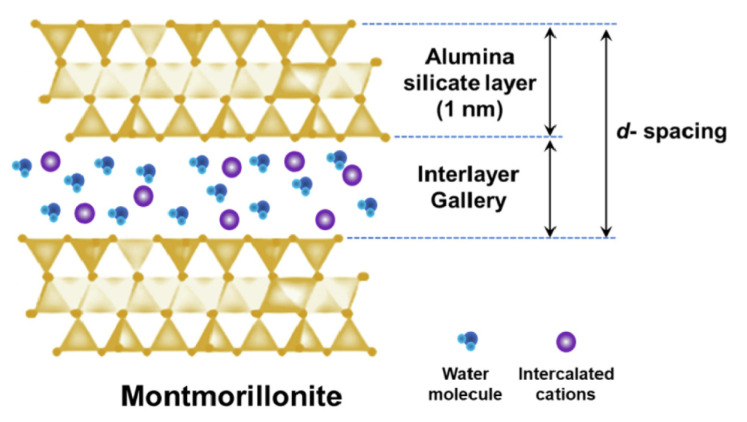
A schematic diagram of the model of one-layer MMT. Reproduced with permission from Ref. [7], Copyright 2022 Elsevier B.V.

The catalysis of inorganic catalysts is that there are many reaction sites on the surface to promote the process of protonation and deprotonation. While a new catalytic role was proposed by Kim et al. A high-performance Fe_2_O_3_@GO catalyst existed due to the synergistic effect of Fe_2_O_3_ nanoparticles and two-dimensional graphene oxide structure. The bound water on the surface of hematite contributed to the formation of a large amount of compact ice-like water which is tetrahedral coordination water with highly interconnected networks and weak O-H bonds at the interface between the bulk water and the Fe_2_O_3_@GO catalyst, which increased the rate of water dissociation by lowering the activation potential barrier [100]. Recently, Eswaraswamy et al. discovered a montmorillonite (MMT) nanoclay with a special spatial structure, which consists of many layers including octahedral alumina sheets located between two tetrahedral silicon sheets [7] (see Figure 20). In the MMT nanoclay, the alumina-silicate layers that are prone to isostructural substitution and the available interface layer channels created charged sites that aided in the protonation–deprotonation step of water molecules and lowered the water dissociation potential. Comparisons of the above interface layer catalysts are shown in Table 1.

For the catalysts themselves, the preparation of porous materials has been a hot research topic. Porous materials have excellent properties such as increased specific surface area, enhanced mass transport and diffusion, and increased active sites [102]. These all speed up the catalytic process. For the catalysts of bipolar membrane interfacial layer, the porous catalyst improved the chance of water molecules entering the bipolar membrane intermediate layer [59], replenished the water needed for hydrolysis of the depleted layer in time, and reduced the occurrence of interface layer delamination. Therefore, the application of porous catalysts to bipolar membranes is of far-reaching significance. For example, as a new type of porous material, metal-organic framework materials have the advantages of large specific surface area, high porosity, adjustable pore size and chemical modification [77,103,104]. It played an active role in promoting the water dissociation of bipolar membrane and reducing the transmembrane voltage. Similar to MOFs, covalent organic frameworks (COFs) are organic porous crystalline materials with the advantages of low density, high specific surface area, and easy modification after synthesis [105,106]. Using porous materials as an intermediate layer catalyst may have a similar or surpassing effect. The pore morphology is controlled by modulating the pore structure of the catalyst (e.g., pore size, length, etc.) to further change the performance of the bipolar membranes. Nanostructures of catalysts are mainly used to increase the surface area and the number of active sites, and nanoconfinement has recently attracted a lot of attention from researchers in terms of affecting electrocatalytic performance. Mesoporous membranes and nanoparticles are the most common structures used in experimental studies. These materials with interconnected pores can achieve selective permeation of substances by controlling the size of the pore channels. Therefore, the pore size is designed according to the type of selected substance to exclude other substances [107]. Using the knowledge of nanoconfinement systems as a means to design higher surface area and high performance electrocatalytic materials has profound effects for the field of electrochemistry.

## 5. Outlook and Thinking

By reading the literature related to interface layer catalysts, many researchers at home and abroad mainly focus on modifying the interface layer of bipolar membranes to reduce the resistance of bipolar membranes and improve water dissociation efficiency. However, there are not many studies on the water dissociation mechanism of the intermediate layer, and the study is not thorough. Moreover, there is a lack of studies on membranes under extreme conditions, such as high temperature and acid-base environments. Therefore, researchers should not only focus on catalysts to accelerate water dissociation and reduce membrane resistance but also comprehensively discuss the performance and applications of bipolar membranes in the future. Mainly reflected in the following aspects:

(1) Further explore the water dissociation mechanism of the interface layer, which can provide a new way for the research and development of materials.
(2) Research on bipolar membranes resistant to high temperature and acid and base. The existing bipolar membranes are difficult to meet the requirements of bipolar membrane electrodialysis in industrial production, and it is necessary to further develop new membrane materials to improve the lifespan of bipolar membranes and reduce energy consumption.
(3) Improve the preparation method of the membranes, increase the adhesion between the bipolar membranes and keep the surface of the prepared membranes smooth and uniform. This can reduce the resistance of the bipolar membranes and reduce the occurrence of delamination of the bipolar membranes when used for a long time.
(4) Investigate the effect of hydrophilic, conductive and catalytic porous materials on water dissociation in bipolar membranes. Porous materials have high surface area and high porosity, which can enhance the mass transfer process. Studying the effect of the structure of pore channels on the performance of bipolar membranes is a direction for the future development of bipolar membrane interface layers.
(5) With the improvement of people’s quality of life and the acceleration of industrialization, it has brought a lot of pollution to the environment, such as domestic wastewater and industrial wastewater. Using BMED technology, inorganic or organic salts could be electrolyzed to generate corresponding inorganic or organic acids. Therefore, BMED technology can also treat wastewater containing various salt components. With the progress of electrodialysis, the metal ions and organic substances contained in the sewage pollute the bipolar membranes, reduce the life of the membrane, and be unfavorable for the reuse of the bipolar membranes. Various process parameters in the BMED process need to be optimized to reduce the cost of the production process. Membrane fouling and energy consumption are problems that need to be faced in the industrialization of bipolar membranes, and solutions are needed to overcome these difficulties in the future.

## Figures and Tables

**Table 1:** U_diss_ and U(100) data for different interface layer catalysts in bipolar membranes. U(100) is the transmembrane voltage at a current density of 100 mA/cm^2^.

Interfacial Layer	U_diss_ (V)	U(100) (V)	Ref.
PEG	3.5	–	[51]
	~1.76	2.50	[95]
BSA	3.22	–	[55]
LYS	2.87	–	[55]
PANI	~1.55	1.87	[56]
KFe[Fe(CN)_6_]	4.25	–	[62]
TiOH	6.93	–	[66]
ZrOH	6.77	–	[66]
SiOH	6.65	–	[66]
GO	–	~1.06	[85]
	0.80	1.20	[1]
	–	1.85	[70]
	3.42	–	[71]
QGO	3.11	–	[71]
PGO	2.83	–	[71]
PGO and QGO	2.39	–	[71]
MoS_2_	~2.50	–	[73]
Fe-MIL-101-NH_2_	~2.60	–	[77]
PAMAM and CrCl_3_	–	2.15	[78]
PAMAM	–	3.04	[78]
CrCl_3_	–	2.50	[78]
dipicolinic acid	0.70	–	[82]
Fe^+3^O(OH)	~1.00	1.10	[93]
P4VP	<0.83	–	[83]
SPEEK	1.87	–	[6]
SPEEK and GO	1.80	–	[6]
Al(OH)_3_	~0.75	~0.93	[92]
	~0.70	–	[91]
	~1.00	–	[90]
Fe(III)@PEI	~1.88	~1.88	[99]
MMT nanoclay	0.73	~0.83	[7]
GO and PVA	~2.10	~5.50	[81]
Fe_2_O_3_@GO	~0.75	0.89	[100]
NiO-IrO_2_ bilayer	~0.75	–	[101]
PEDOT:PSS and PEI	~0.70	~2.5	[3]

## Data Availability

The data presented in this study are available on request from the corresponding author.
